# Mapping Sex Differences in Depression, Anxiety, and Co‐Occurring Symptoms in Adults Under Stress: A Bayesian Network Approach

**DOI:** 10.1155/da/6640438

**Published:** 2026-09-12

**Authors:** Jingchu Hu, Yiting Huang, Marlon Westhoff, Qingzhuo Ren, Stefan G. Hofmann

**Affiliations:** ^1^ Department of Anxiety Disorders, Shenzhen Clinical Research Center for Mental Disorders, Shenzhen Kangning Hospital, Shenzhen, Guangdong, China; ^2^ Affiliated Mental Health Center, Southern University of Science and Technology, Shenzhen, Guangdong, China, sustc.edu.cn; ^3^ Department of Psychology, Philipps-Universität Marburg, Schulstraße 12, 35032, Marburg, Germany, uni-marburg.de

**Keywords:** anxiety, depression, fear, insomnia, PTSD, sex differences, stress, symptoms network

## Abstract

**Background:**

Sex differences in the prevalence and presentation of depression and anxiety are well‐established, yet disparities in the underlying network structure and potential causal relationships between symptoms, particularly under significant stress, remain less understood. Understanding these network differences is crucial for elucidating mechanisms and tailoring interventions. This study explored sex differences in the network structure of depression, anxiety, and co‐occurring symptoms (posttraumatic stress disorder [PTSD] symptoms, insomnia, and fear reactions) in an adult population.

**Methods:**

A cross‐sectional survey was conducted among adults (*N* = 1,191, 684 women, aged 18–40) in Shanghai during a period of significant acute public health stress (pandemic citywide lockdown). Participants reported symptoms of depression (Patient Health Questionnaire [PHQ]), anxiety (Generalized Anxiety Disorder [GAD]), PTSD (intrusion, avoidance), insomnia (Insomnia Severity Index [ISI]), and fear reactions. Partial correlation networks examined overall symptom connectivity. Bayesian network (BN) analysis generated directed acyclic graphs (DAGs) to infer potential directional dependencies. Analyses were conducted by sex.

**Results:**

Distinct sex‐specific symptom network patterns emerged. Anxiety and intrusion symptoms were most central for women, while depression was most central for men. Overall network connectivity was significantly higher in women. Critically, BN analysis identified a more complex architecture of probabilistic dependencies in women, evidenced by a greater number of directed edges compared to men. The men’s DAG indicated a relatively sparse structure with fewer directional pathways between symptoms.

**Conclusions:**

Men and women exhibited distinct distress symptom network structures under acute stress. Specifically, anxiety and intrusion emerged as central nodes in women, indicating that they may represent promising intervention targets to prevent symptom escalation, while depressive symptoms were more central to men. These findings highlight the importance of sex‐sensitive psychological first aid during public health crises and establish an empirical foundation for the development of sex‐informed, process‐oriented interventions in future research.

## 1. Introduction

The outbreak of the COVID‐19 pandemic has not only posed a significant global public health stress but has also led to a pervasive psychological crisis affecting individuals worldwide [1, 2]. The lockdown measures may disrupt millions of people’s normal routines, social interactions, and economic activities, leading to increased stress, anxiety, depression, and other psychological problems [3, 4]. While epidemiological research has thoroughly documented the increases in psychiatric symptoms during this period, most studies have concentrated on the severity or prevalence of individual disorders [5, 6]. However, mental disorders do not exist in isolation; rather, their symptoms co‐occur and overlap across diagnostic boundaries, forming complex and mutually reinforcing systems [7]. Despite this recognition, there remains a lack of understanding of how symptoms such as depression, anxiety, insomnia, and posttraumatic stress disorder (PTSD) are interconnected and potentially maintain one another under acute stress, particularly in the working‐age adult population (ages 18–40). Furthermore, it remains unclear whether the structure of these symptom interrelations differs between men and women. Addressing these gaps is essential as a more fine‐grained understanding of sex‐specific central symptoms and potential symptom dynamics could inform process‐focused interventions. Treatment effect sizes for specific disorders, such as anxiety‐related disorders [8] or depression [9], have remained largely unchanged over recent decades, underscoring the need to identify sex‐specific central symptoms and their dynamics, which may help refine strategies to more effectively disrupt distress cycles.

### 1.1. The Shanghai Lockdown and the Sociocultural Context of Psychological Distress

Shanghai, one of the most populous and economically developed cities in mainland China, underwent one of the country’s longest and most stringent citywide lockdowns from April to June 2022. Prolonged home confinement, mobility restrictions, and economic uncertainty created a context of sustained acute stress for residents [10, 11]. Working‐age adults (ages 18–40), often described as the “sandwich generation” in Chinese society, faced compounded pressures during this period, including career instability and heightened caregiving responsibilities for both children and older family members [12].

In China’s sociocultural context, these stressors are intertwined with cultural values that emphasize collective responsibility and emotional restraint. Traditional gender role expectations further structure stress exposure. For instance, women often take on disproportionate domestic and caregiving duties, while men frequently face heightened financial pressure amid labor market instability [13]. Emerging cross‐cultural research suggests that sociocultural variables, particularly collectivism versus individualism, fundamentally influence the topological organization of psychopathological symptoms within network models. For instance, a recent study reported substantial cultural heterogeneity in depression and social anxiety symptom networks, demonstrating how collectivistic orientations specifically shape the interconnectivity between shame‐related and mood symptoms [14]. Despite the growing focus on pandemic‐related mental health issues, there is limited empirical evidence on the internal architecture of stress‐related symptoms among working‐age adults in the specific sociocultural context of the Shanghai lockdown. Furthermore, it has not been systematically examined whether such network configurations differ between men and women in this high‐stress setting. Understanding potential differences in symptom network configuration may provide a more nuanced perspective on how psychological distress is structured within this unique sociocultural context.

### 1.2. Acute Stress and Symptom Co‐Occurrence

Acute public health crises, such as the COVID‐19 pandemic and associated lockdowns, are consistently linked to increased rates of depression, anxiety, insomnia, and PTSD symptoms [15, 16]. These symptoms frequently co‐occur rather than occurring in isolation, suggesting complex patterns of interrelation under conditions of sustained stress. Although global meta‐analyses have documented increases in the prevalence of individual disorders during the pandemic [17, 18], such epidemiological approaches generally conceptualize symptoms as independent or aggregated outcomes.

However, mounting evidence indicates that psychological symptoms are not independent entities but part of dynamic systems in which changes in one symptom can spread throughout the network, reflecting interdependencies among manifestations of distress [19]. For instance, sleep disturbances, affective symptoms, and hyperarousal frequently co‐occur in stress‐related conditions, underscoring the importance of examining their structural organization rather than their overall severity alone [20]. A critical gap in current research is the limited attention given to how these stress‐related symptoms are organized at the network level within specific populations. Furthermore, although sex differences in mean symptom levels have been widely reported, considerably less is known about whether the configuration of symptom interrelations differs between men and women under acute stress.

### 1.3. Sex Differences in Stress‐Related Symptoms

Women have consistently reported higher levels of anxiety, depression, and posttraumatic stress symptoms during public health crises [21]. These disparities are commonly interpreted in light of differential exposure and social roles, including disproportionate caregiving responsibilities, increased domestic labor, and heightened vulnerability to economic stressors [22, 23]. For example, during the 2022 Shanghai lockdown, women reported greater exposure to intimate partner violence and financial strain [24]. Conversely, prevailing social norms may discourage men from openly expressing emotional distress or seeking psychological support, potentially influencing symptom reporting patterns [25]. Importantly, such differences between women and men should not be interpreted as exclusively reflecting biological sex. Because many studies, including the present one, classify participants by sex without directly measuring gender identity, gender roles, or gendered stress exposures, the observed group differences may reflect a combination of sex‐related biological processes and gender‐related social mechanisms.

However, mean‐level differences do not necessarily imply differences in how symptoms are structurally organized. Network psychopathology offers a powerful framework with which to examine these intrinsic dynamics, moving beyond aggregated severity scores to explore patterns of associations among individual symptoms [19]. To date, research exploring sex‐ or gender‐related variations in symptom networks following traumatic events has yielded mixed results. For instance, studies conducted among disaster‐exposed adults in Latin America [26] and survivors of the 2011 Oslo terrorist attack [27] found that while females reported higher symptom levels, there were no significant sex differences in the global network structure or connectivity strength. These findings suggest that elevated symptom severity does not always correspond to large‐scale structural differences in symptom interrelations. Nevertheless, a recent network analysis of Chinese adults during the COVID‐19 pandemic identified differences in specific central PTSD symptoms and connection patterns between men and women [28].

Despite these insights, the existing literature remains limited in several aspects: most studies focus on chronic PTSD symptoms in isolation, rely on Western populations, or exclusively examine clinical samples. Under the unique, large‐scale stress of a metropolitan lockdown in a collectivistic society, it remains unclear whether a more densely connected and reactive system—where symptoms like insomnia more easily trigger a cascade of anxiety and depression—emerges differently between women and men. Understanding these distinct “symptom‐to‐symptom” pathways within a broader constellation of symptoms is crucial to developing tailored interventions that target the most influential “hub” nodes for each sex‐disaggregated group.

### 1.4. A Dual‐Network Perspective on Symptom Structure

Network analysis offers a novel approach to conceptualize psychopathology by identifying interrelationships among symptoms and reveals the dynamics that maintain psychological distress [7, 19, 29, 30]. In these network models, nodes represent individual symptoms, while edges represent the statistical relationships between them. Identifying central symptoms—those with high connectivity or influence—is crucial, as these “hub” nodes may serve as primary targets for clinical intervention [19, 30]. However, standard undirected networks (e.g., Gaussian graphical models [GGM]) primarily capture associations (edge weights) and relative importance (centrality) without clarifying the potential direction of influence between symptoms.

Bayesian networks (BNs) extend this approach by modeling probabilistic conditional dependencies, which are represented as directed acyclic graphs (DAGs) [31]. In a DAG derived from cross‐sectional data, a directed edge from symptom A to symptom B indicates that, after controlling for all other nodes in the network, the state of symptom A provides predictive information about the state of symptom B. Although BNs based on cross‐sectional data cannot definitively establish temporal causality, they allow for the inference of potential directional pathways and dependencies that are statistically consistent with the observed data structure.

Integrating undirected partial correlation networks (to identify core associations and connectivity strength) with directed BNs (to explore potential influence pathways and complex dependencies) provides a more comprehensive understanding of symptom interplay than either method alone [32]. This dual‐network approach is particularly suited for elucidating how sex‐specific configurations of depression, anxiety, and insomnia are organized and maintained under acute stress.

### 1.5. The Present Study

In the present study, we applied two complementary network modeling approaches to investigate sex‐based differences in the structure of stress‐related symptoms among adults in Shanghai during a period of large‐scale public health confinement. First, we estimated a GGM [33] to map partial correlations among symptoms of depression, anxiety, and insomnia and compared global network properties between men and women. Although prior research has investigated sex differences in PTSD symptom networks following discrete traumatic events, relatively little is known about whether broader stress‐related symptom constellations differ by sex under large‐scale stress conditions.

Second, to gain initial insights into possible directional dependencies among stress‐related symptoms, we implemented a BN analysis by estimating a DAG [31]. This nuanced understanding is essential for providing clinical practitioners with robust empirical evidence and facilitating the development of tailored intervention strategies that address the specific symptom structures activated during major public health crises.

Based on previous findings indicating sex differences in symptom expression and network connectivity following stressful events [24–26], we hypothesized that although the global network architecture might show some similarities, specific node centralities and edge patterns would differ between men and women, reflecting distinct sex‐specific patterns of psychological distress. Moreover, we hypothesized that the application of BN modeling would enable the identification of directional dependencies among stress‐related symptoms, thereby providing insights into potential pathways through which depressive, anxious, and insomnia symptoms may reinforce one another in sex‐specific ways.

## 2. Method

### 2.1. Study Design, Participants, and Procedure

From April 18 to July 12, 2022, a cross‐sectional survey was conducted in Shanghai City during the lockdown period. The study was approved by the Institutional Review Board of Shenzhen Kangning Hospital (2020‐3‐20‐2). Data for the present study were collected using online forms hosted on the Wenjuanxing web‐based public platform (https://www.wjx.cn). These forms were distributed among potential respondents from the platform panel. The platform implemented measures such as ID, email, and location verification to maintain the quality of responses. Informed consent was obtained electronically at the beginning of the online questionnaire. Participants were presented with a detailed information sheet outlining the purpose of the study, procedures involved, and their rights. Only those who provided electronic and written consent were able to proceed with the survey. The study employed specific inclusion criteria. Participants were required to (1) be aged above 18 and not exceeding 40 years, (2) have been residing in Shanghai City during the lockdown period, and (3) complete the entire questionnaire. Upon completing the survey, participants were compensated with a reward through online payment on WeChat. Each participant received 2 CNY, approximately equivalent to 0.3 USD. Since this study was an observational, cross‐sectional survey conducted on a nonclinical sample and involved no clinical intervention, trial registration was not required under the current International Committee of Medical Journal Editors (ICMJE) guidelines.

The survey yielded a total of 1840 completed responses. However, 649 participants did not meet the inclusion standards due to the following reasons: (1) 213 participants fell below the age range (below 18) or exceeded it (above 40); (2) 395 participants were not residing in Shanghai City during the lockdown period; and (3) 41 participants did not successfully respond to the trap/attention questions, designed to ensure response attentiveness (e.g., correctly identifying the capital city in China). Participants were grouped based on their self‐reported sex. No separate measures of gender identity, gender roles, or gender‐related social exposures were collected. For the purpose of the current study, the final sample consisted of 1191 Chinese adult participants aged between 18 and 40 years, with 57.43% identified as women. Only complete questionnaires were included in the analysis; therefore, no imputation of missing data was performed.

### 2.2. Measurement

#### 2.2.1. The Fear of COVID‐19 Scale (FCV‐19S)

The Fear of COVID‐19 Scale (FCV‐19S) [34] is a timely self‐reported questionnaire designed to measure the level of fear during the COVID‐19 pandemic. It focuses on two aspects: the perceptions of fear in fear‐thinking and the physical response of fear. This study used the Chinese version of the Fear of COVID‐19 pandemic scale [35]. The scale consists of 7 items assessed on a 5‐point Likert scale ranging from 1 (strongly disagree) to 5 (strongly agree). The Chinese FCV‐19S has a two‐dimensional structure: physical reactions refer to the physiological experience of fear (e.g., “My hands become clammy when I think about coronavirus‐19”), and fear thoughts refer to the subjective emotional experience of fear (e.g., “I am most afraid of coronavirus‐19”). A higher score refers to a greater fear of COVID‐19. In this study, Cronbach’s *α* for fear thoughts was 0.84 for both women and men; for physical reactions, it was 0.81 for women and 0.79 for men.

#### 2.2.2. The Impact of Event Scale (IES)

The IES [36] was developed to assess PTSD, and the IES with modifications for COVID‐19 (IES‐COVID‐19) was used in the current study [37] to evaluate trauma‐related stress symptoms associated with the impact of the COVID‐19 pandemic. The scale consists of 15 items measured on a 4‐point Likert scale, ranging from 1 (not at all), 2 (seldom), 3 (sometimes), to 4 (often). The IES‐COVID‐19 includes two subscales: Intrusion (e.g., “Pictures about it popped into my mind”) and avoidance (e.g., “I stayed away from reminders of it”). Higher scores indicate a stronger PTSD symptom. It should be noted that while the IES is a widely validated measure of stress response, it was developed based on DSM‐IV criteria and does not fully encompass all DSM‐5 PTSD symptom clusters, such as negative alterations in cognitions and mood. In the present sample, Cronbach’s *α* for intrusion was 0.78 (women) and 0.77 (men) and for avoidance was 0.72 (women) and 0.73 (men).

#### 2.2.3. The Patient Health Questionnaire (PHQ‐9)

The PHQ‐9 [38] is a self‐reported questionnaire used to assess depressive symptoms over the past 2 weeks. This study utilized the Chinese version of PHQ‐9 for the general population [39]. The questionnaire consists of 9 items (e.g., “Feeling down, depressed, or hopeless”) measured on a 4‐point Likert scale from 0 (not at all) to 3 (nearly every day). Higher scores indicate more severe depression. In this study, the internal consistency values were Cronbach’s *α* = 0.84 for men and Cronbach’s *α* = 0.85 for women.

#### 2.2.4. The Generalized Anxiety Disorder Scale (GAD)

The GAD Scale [40] is a self‐reported questionnaire comprising 7 items (e.g., “Not being able to stop or control worrying”) measured on a 4‐point Likert scale, ranging from 0 (not at all) to 3 (nearly every day). It assesses the severity of symptoms related to generalized anxiety disorder over the past 2 weeks. The current study utilized the Chinese version of GAD [41] for the general population. Higher scores on the scale indicate more severe anxiety. In the current study, Cronbach’s *α* values were 0.85 for women and 0.85 for men.

#### 2.2.5. The Insomnia Severity Index (ISI)

The ISI [42] is a self‐reported questionnaire designed to measure the severity of nighttime and daytime insomnia components. Participants rated 7‐items (e.g., “Difficulty falling asleep”) on a 5‐point Likert scale ranging from 0 to 4 (0 = not at all/very satisfied to 4 = very severe/very dissatisfied), with total scores ranging from 0 to 28. The Chinese version of the ISI [43] was employed in the study. Higher scores on the scale indicate more severe insomnia. In this study, the internal consistency values were Cronbach’s *α* = 0.88 for women and 0.88 for men.

### 2.3. Data Analysis

The study employed independent *t*‐tests and chi‐squared tests, along with descriptive analyses, to examine the differences in demographic and clinical variables between women and men. Continuous variables are presented as the mean (*M*) and standard deviation (SD), and categorical variables as percentages and frequencies. The test level was two‐sided with a significance level of *p* < 0.05.

Due to the significant sex disparities observed in education and occupation (Table 1), and given the established relevance of these sociodemographic factors for mental health, these variables were included as covariates in a sensitivity analysis to ensure the robustness of the primary network estimations. This ensured that the observed structural differences were not artifacts of sociodemographic imbalances. Then, network structures were estimated separately for women and men to investigate the differences in connectivity between the network structures. All analyses were performed using IBM SPSS (Version 26.0), the statistical programming language R (Version 4.2.1), and a variety of R packages. Power analysis was performed using the Powerly package in R to estimate the recommended sample size for a GGM with seven nodes in each sex‐specific network. With a target sensitivity of 0.6 and power of 0.8, the recommended sample size was 168, with a 95% interval of 150–187 (Figure S1) [44].

**Table 1:** Sociodemographic characteristics and clinical variable scores of the study population (*N* = 1191), disaggregated by sex.

Characteristics		Women (*n* = 684)	Men (*n* = 507)	Diff	
		Mean/*n* (SD/%)	Mean/*n* (SD/%)	*t*/chisq	*p*
Demographics					
Age n (%)		—	—	5.24	0.07
18–25		210 (30.70)	126 (24.90)	—	—
26–30		216 (31.60)	181 (35.70)	—	—
31–40		258 (37.70)	200 (39.40)	—	—
Marital status n (%)		—	—	0.80	0.37
Unmarried		309 (45.20)	215 (42.40)	—	—
Married or divorced		375 (54.80)	292 (57.60)	—	—
Education level n (%)		—	—	7.74	0.02 ^∗^
Senior high school or lower		103 (15.10)	106 (20.90)	—	—
Bachelor’s degree		498 (72.80)	335 (66.10)	—	—
Master’s degrees or higher		83(12.10)	66(13.00)	—	—
Occupation n (%)		—	—	10.55	0.01 ^∗^
Employee/employer		513 (75.00)	391 (77.10)	—	—
Student		114 (16.70)	60 (11.80)	—	—
Unemployed		37 (5.40)	45 (8.90)	—	—
Other		20 (2.90)	11 (2.20)	—	—
Income (¥/year) n (%)		—	—	1.36	0.85
< 10,000		110 (16.10)	91 (17.90)	—	—
80,001–150,000		190 (27.80)	141 (27.80)	—	—
150,001–800,000		344 (50.30)	245 (48.30)	—	—
> 800,001		29 (4.30)	24 (4.70)	—	—
Unknown		11 (1.60)	6 (1.20)	—	—
Main variables mean (SD)					
Node name	Item content/overall scales	—	—	—	—
PHQ	Patient Health Questionnaire	7.78 (5.09)	7.89 (5.26)	−0.34	0.73
GAD	Generalized Anxiety Disorder	6.40 (4.30)	6.20 (4.34)	0.80	0.42
ISI	Insomnia Severity Index	8.61 (5.39)	8.65 (5.35)	−0.13	0.90
FCV	Fear of COVID‐19 Scale	18.84 (6.32)	17.66 (6.31)	3.19	0.001 ^∗∗^
FCVft^a^	Fear thoughts^a^	12.16 (3.97)	11.28 (4.14)	3.67	<0.001 ^∗∗∗^
FCVpr^a^	Physical response^a^	6.68 (2.89)	6.38 (2.77)	1.85	0.06
IES	Impact of Event Scale	26.87 (12.80)	26.30 (12.94)	0.75	0.45
AVO^b^	Avoidance^b^	14.94 (7.44)	14.98 (7.73)	−0.09	0.92
INTRU^b^	Intrusion^b^	11.93 (6.80)	11.32 (6.71)	1.54	0.12

*Note: N* = 1191. Socioeconomic status (SES) was assessed via education, occupation, and annual income. Continuous variables are presented as mean (SD), and categorical variables as *n* (%). All psychiatric symptoms were assessed in relation to the Shanghai lockdown.

^a^FCVft and FCVpr are subscales of the FCV‐19S.

^b^INTRU and AVO are subscales of the IES‐COVID‐19.

^∗^ *p*  < 0.05.

^∗∗^ *p*  < 0.01.

^∗∗∗^ *p*  < 0.001.

#### 2.3.1. Network Estimation

We estimated a separate cross‐sectional network model for both women and men to examine the relationships between the fear of COVID‐19 and individual symptoms of anxiety, depression, insomnia, and PTSD. The GGM was visualized using the *qgraph* package [45]. In the visualized networks, the color of the edge reflects the direction of association (green edges indicate positive association, while red edges represent negative association between the nodes). The thickness of the edge reflects the strength of the associations between the nodes. The Extended Bayesian Information Criterion (EBIC) was applied to achieve an optimal and sparse estimate of the partial correlation matrix, with the tuning parameter set to 0.50 to balance selectivity and specificity [46]. The combination of GLASSO and EBIC has demonstrated sensitivity in detecting edges in sparse graphs with large sample sizes (i.e., *n* > 250) [33].

To evaluate the relative importance of each node in the network, we computed four centrality indices: strength, expected influence, betweenness, and closeness [47]. These centrality indices were calculated using the R package *qgraph* [48]. Strength reflects the sum of the absolute edge weights connected to a node and thus indicates how strongly a node is directly connected to the rest of the network. Betweenness quantifies how frequently a node lies on the shortest path between two other nodes, indicating its efficiency in connecting different parts of the network. Closeness represents the average distance from a given node to all other nodes, indicating its proximity to other nodes. Higher values on each index indicate greater centrality and, in principle, a higher potential influence within the symptom network.

Then, covariates were controlled in two networks without making the assumption that they form part of the causal system [49]. Using the *mgm* package, we estimated mixed graphical models that can accommodate different variable types. Correlation analysis was performed to estimate whether the covariates significantly affect the network model [50].

Moreover, the predictability of each node was estimated using the R package *mgm*. Predictability measurements indicate the amount of variance in a node that is explained by all other nodes in the network [50].

#### 2.3.2. Network Stability

Furthermore, we assessed the accuracy and stability of centrality indices and edge weights using bootstrap procedures implemented in the *bootnet* package [33].

To assess the stability of node centrality estimates, we conducted case‐drop bootstrapping and computed the correlation stability coefficient (CS‐coefficient) [33]. The CS coefficient represents the maximum proportion of cases that can be removed from the data while maintaining an average correlation of *r* > 0.70 between the centrality indices of the full dataset and those in the subsetted dataset with a 95% probability [33, 44]. A CS coefficient above 0.25 is typically considered minimally acceptable, whereas values of 0.50 or higher indicate good stability [33]. To assess edge accuracy, we calculated nonparametric bootstrapped 95% confidence intervals (CIs). Narrower CIs indicate greater precision in the estimation of edge strengths, whereas wider intervals suggest that specific edge weights should be interpreted with caution [42].

#### 2.3.3. Sex Comparison of Network Characteristics

Finally, we used the R package *NetworkComparisonTest* (*NCT*) to assess the sex differences between women’s and men’s symptom networks. The network structure invariance test and edge invariance test examined the maximum difference in the global network structure and edge weights in the two networks by comparing the distributions of edges, and the global strength invariance test was run to compare the summed absolute value of all the edges in two networks [51]. This permutation‐based test randomly reshuffles individuals between the two groups (women and men) multiple times (5000 times) and compares the subgroups’ networks [51].

#### 2.3.4. BN (DAG)

We additionally estimated a DAG using BN analysis to explore conditional independence relationships and probabilistic directional dependencies among the nodes in the cross‐sectional data [29]. In these models, a directed edge from node A to node B indicates that the activation of A predicts the activation of B more strongly than the reverse direction under the assumption that no relevant variables are omitted. Although DAGs cannot establish temporal precedence or full causal claims with cross‐sectional data, they can provide useful hypotheses about potential causal pathways when interpreted alongside the GGM results.

Following previous work, we used the hill‐climbing algorithm implemented in the R package *bnlearn* to estimate the DAGs [52]. This score‐based search procedure iteratively adds, removes, and reverses edges to optimize a goodness‐of‐fit index (i.e., Bayesian Information Criterion [BIC]) [52]. To enhance the robustness of the DAG, we adopted the bootstrap approach (5000 bootstrap samples with replacement) to obtain the final DAG structure [53]. Edges that appeared more frequently between nodes were assigned higher scores, indicating strength and directional probability. Then, the network was averaged and retained only the relationships present in 85% of the models produced. A significance threshold of 0.85 was enforced to depict only the most reliable edges in the model [53]. Last, the inclusion of directional edges in the final DAG hinged upon the presence of edges pointing in the same direction in 50% or more of the 5000 bootstrapped DAGs. In the visualized DAG, edge thickness signifies the improvement in the BIC values upon inclusion of the specific edge.

## 3. Results

### 3.1. Characteristics of the Study Population

Table 1 presents the socio‐demographic characteristics and clinical variable scores of the sample according to sex. Of the participants, 57.43% were women, and 42.57% were men. Both women and men were evenly distributed across three age periods: 18–25, 26−30, and 31–40. A slightly higher proportion of men were married or divorced (57.6%) compared to women (54.8%). Women were more educated, as 84.9% (vs. 79.1% of men) had a bachelor’s degree or higher. A higher proportion of women and men were employees/employers, with 75.0% and 77.1%, respectively.

Women were more likely to report lower scores for depression (PHQ‐9) and insomnia (ISI) while reporting higher scores for anxiety (GAD) and PTSD (IES‐COVID‐19), though the differences were not significant. Fear of COVID‐19 (FCV‐19S) was significantly higher in women than in men (*p* = 0.001), driven primarily by the fear thoughts dimension (*p* < 0.001), while the physical response dimension showed only a marginal difference (*p* = 0.06).

### 3.2. Network and Centrality Estimation in Women and Men

**Figure 1:**
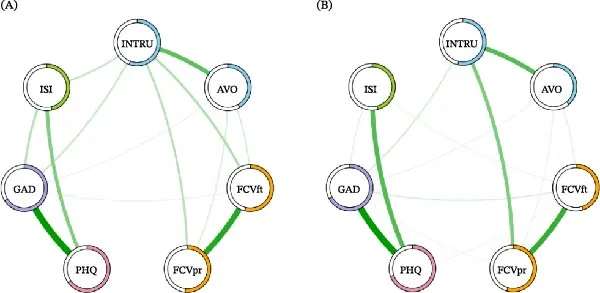
(A) Estimated network structure in women’s group (*n* = 684). (B) Estimated network structure in men’s group (*n* = 507). Lines represent edges, and circles represent variables; green lines indicate positive LASSO‐regularized partial correlations; the thickness of the lines indicates the weights of the edges. Ring‐shaped pies represent predictability (the rings around the nodes indicate the proportion of variance explained by its intercorrelations with the other nodes in the network). The color of the nodes denotes their community belonging. PTSD_Intrusion (INTRU), PTSD_Avoidance (AVO), fear thoughts (FCVft), physical reactions (FCVpr), depression (PHQ), anxiety (GAD), insomnia (ISI).

Graphical LASSO networks for women and men are shown in Figure 1, with edge weights summarized in Table S1 and Table S2. The predictability of symptoms for women and men is shown as ring‐shaped pie charts in Figure 1. Mean predictability was 0.54 in women and 0.53 in men, indicating that the average variance in each node could be accounted for by its neighboring nodes.

**Figure 2:**
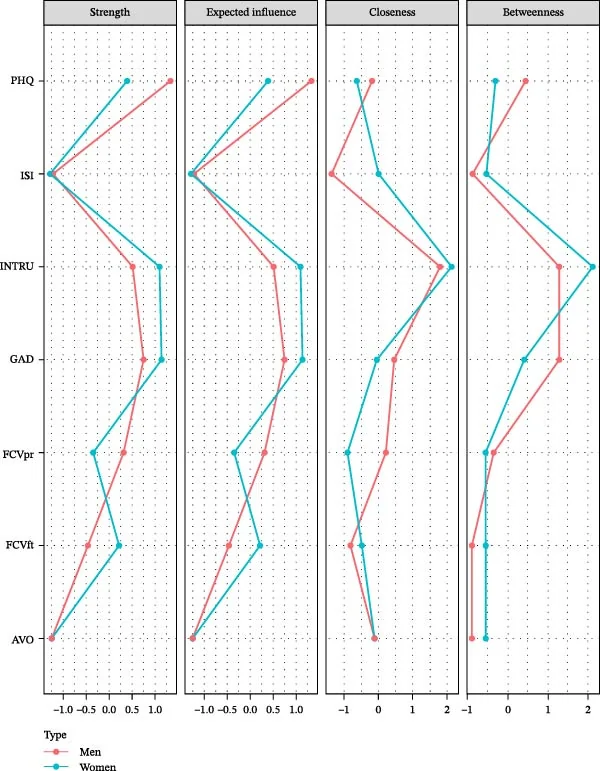
Standardized centrality indices of the network for women and men (strength, expected influence, betweenness, and closeness). A higher centrality index for a node implied a more central relationship. PTSD_Intrusion (INTRU), PTSD_Avoidance (AVO), fear thoughts (FCVft), physical reactions (FCVpr), depression (PHQ), anxiety (GAD), insomnia (ISI).

There were seven nodes and 21 edges (7 × (7–1)/2) in both networks. Of the edges, 61.90% (13 edge connections) and 42.86% (9 edge connections) were nonzero weight in women’s and men’s networks, respectively, indicating that most psychological distress symptoms are closely intercorrelated with each other. Because education level (*p* = 0.02) and occupation (*p* = 0.01) differed significantly between sexes (Table 1), these variables were included as covariates in a sensitivity analysis. This ensured that the network structures reflected sex‐specific symptom dynamics rather than sociodemographic variances. Following previous research [47, 52], the network model and structure indexes for women and men were re‐estimated. The edge‐weight matrices of the original and covariate‐adjusted networks correlated highly (women: *r* = 1, *p* < 0.001; men: *r* = 1, *p* < 0.001), suggesting that sociodemographic differences did not substantially influence the network model. Figure 2 presents the centrality metrics (strength, expected influence, betweenness, and closeness) of the graphical LASSO networks. A higher centrality index for a node implies a more central relationship in the network and close linkage to other symptoms [53]. In the network for women, anxiety presented the comparatively highest strength and expected influence, followed by intrusion, depression, fear of thoughts, physical reactions, avoidance, and insomnia. The comparatively strongest edges were PHQ‐GAD (0.61), followed by FCVft‐FCVpr (0.52), AVO‐INTRU (0.38), and PHQ‐ISI (0.30) (refer to Table S1 for the weight of each connection in the women’s network). However, no edge weights between PHQ‐INTRU, PHQ‐AVO, PHQ‐FCVft, PHQ‐FCVpr, GAD‐FCVpr, ISI‐AVO, ISI‐FCVft, and ISI‐FCVpr were found in the women’s model.

In men, depression presented the comparatively highest strength and expected influence, followed by anxiety, intrusion, physical reactions, fear thoughts, avoidance, and insomnia (Figure 2). Intrusion and anxiety showed the comparatively greatest closeness and betweenness. Similar to the network for women, in the network for men (Table S2), the comparatively strongest edges were between PHQ‐GAD (0.63), followed by FCVft‐FCVpr (0.51), AVO‐INTRU (0.42), and PHQ‐ISI (0.42). Notably, an edge was found in the network for men between FCVpr and INTRU (0.32), which was less connected in the network for women (0.17). However, no edge weights between PHQ‐INTRU, PHQ‐AVO, PHQ‐FCVft, PHQ‐FCVpr, GAD‐FCVpr, ISI‐INTRU, ISI‐AVO, ISI‐FCVft, and ISI‐FCVpr were found in the men’s model.

Network stability was evaluated using case‐dropping bootstrapping (with 5000 bootstrap samples) to calculate the CS coefficients. The stability of edge weights was excellent, reaching the maximum tested threshold for both networks (women: CS = 0.75 and men: CS = 0.75). Node strength centrality also demonstrated high stability (women: CS = 0.67 and men: CS = 0.75), exceeding the preferred 0.50 threshold. As is common in psychological networks, closeness (women: CS = 0.52; men: CS = 0.21) and betweenness (women: CS = 0.36; men: CS = 0.21) centralities exhibited lower stability. These indices are therefore not interpreted for the men’s network. Furthermore, bootstrapped 95% CIs for edge weights were narrow and nonzero for most edges in both networks, confirming their accuracy (refer to Figures S2 and S3). Bootstrapped difference tests indicated that most comparisons among edge weights, as well as node strength centrality, were statistically significant and stable (refer to Figures S4–S7), supporting the overall reliability of the network models.

### 3.3. Sex Differences in Global and Local Connectivity of Networks

The global strength invariance test showed that the network strength was significantly stronger in women (global strength = 2.96) than in men (global strength = 2.67, *p* = 0.02). The network invariance test indicated significant network structure differences between sexes (*p* = 0.02). Table 2 shows the results of the edge invariance test between sexes. The results revealed a significantly stronger edge in women between INTRU‐FCVft (*p* = 0.003). Conversely, the edges between INTRU‐FCVpr (*p* = 0.008) was stronger in men.

**Table 2:** Edge invariance test between women and men.

Edge between		Edge‐weight (women)	Edge‐weight (men)	*p*‐Value
INTRU^a^	AVO^a^	0.38	0.42	0.43
INTRU^a^	FCVft^b^	0.20	0.00	<0.01 ^∗∗^
AVO^a^	FCVft^b^	0.11	0.08	0.72
INTRU^a^	FCVpr^b^	0.17	0.32	<0.01 ^∗∗^
AVO^a^	FCVpr^b^	0.10	0.00	0.50
FCVft	FCVpr^b^	0.52	0.51	0.68
INTRU^a^	PHQ	0.00	0.00	1.00
AVO^a^	PHQ	0.00	0.00	1.00
FCVft^b^	PHQ	0.00	0.00	1.00
FCVpr^b^	PHQ	0.00	0.00	1.00
INTRU^a^	GAD	0.14	0.13	0.85
AVO^a^	GAD	0.06	0.00	0.82
FCVft^b^	GAD	0.05	0.08	0.61
FCVpr^b^	GAD	0.00	0.00	1.00
PHQ	GAD	0.61	0.63	0.57
INTRU^a^	ISI	0.15	0.00	0.06
AVO^a^	ISI	0.00	0.00	1.00
FCVft^b^	ISI	0.00	0.00	1.00
FCVpr^b^	ISI	0.00	0.00	1.00
PHQ	ISI	0.30	0.42	0.06
GAD	ISI	0.18	0.09	0.14

*Note:* FCVft, fear thoughts; FCVpr, physical reactions; PHQ, depressive symptoms; GAD, anxiety symptoms; ISI, insomnia symptoms.

Abbreviations: AVO, avoidance; INTRU, intrusion.

^a^INTRU and AVO are subscales of the IES‐COVID‐19.

^b^FCVft and FCVpr are subscales of the FCV‐19S.

^∗^ *p* < 0.05

^∗∗^ *p* < 0.01

^∗∗∗^ *p* < 0.001

Table 3 presents the results of the node invariance test between sexes. The strength centralities of fear thoughts were significantly higher for women (*p* = 0.03), and depression was significantly higher for men (*p* = 0.04).

**Table 3:** Node invariance test between women and men.

Node	Strength (women)	Strength (men)	*p*‐Value
INTRU^a^	1.03	0.87	0.07
AVO^a^	0.64	0.50	0.25
FCVft^b^	0.88	0.67	0.03 ^∗^
FCVpr^b^	0.79	0.83	0.61
PHQ	0.91	1.05	0.04 ^∗^
GAD	1.04	0.92	0.24
ISI	0.63	0.50	0.10

*Note:* FCVft, fear thoughts; FCVpr, physical reactions; PHQ, depressive symptoms; GAD, anxiety symptoms; ISI, insomnia symptoms.

Abbreviations: AVO, avoidance; INTRU, intrusion.

^a^INTRU and AVO are subscales of the IES‐COVID‐19.

^b^FCVft and FCVpr are subscales of the FCV‐19S.

^∗^ *p* < 0.05.

### 3.4. DAG (Bayesian Approach)

**Figure 3:**
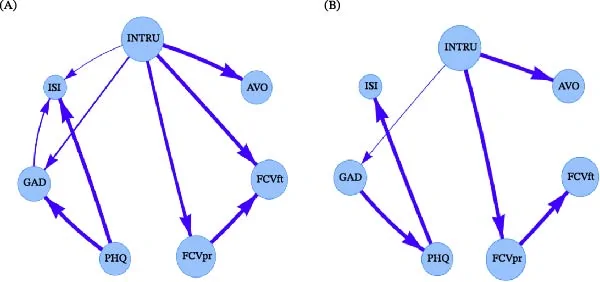
Directed acyclic graphs (DAGs) for (A) women’s group (*n* = 684) and (B) men’s group (*n* = 507). Edge thickness denotes the bootstrap strength (proportion of bootstrap samples in which the directed edge appears), with thicker edges indicating higher stability. Nodes: INTRU = PTSD intrusion, AVO = PTSD avoidance, FCVft = fear of COVID‐19 fear thought, FCVpr = fear of COVID‐19 physical reaction, PHQ = depression, GAD = anxiety, ISI = insomnia.

These directional patterns were broadly consistent with the undirected network structure, particularly regarding the central role of intrusion in women and affective symptoms in men. Figure 3 illustrates the inferred directional pathways within the psychological distress networks. The women’s network contained a greater number of influential relationships, displaying nine directed arcs, whereas six arcs were observed within the men’s network. Table 4 presents the estimated strength and direction of relationships between the men’s and women’s networks. Within the women’s network, INTRU emerged as a primary upstream node, exhibiting directional paths toward AVO (Strength (*S*) > 0.99, Direction (*D*) = 0.59), FCVft (*S* > 0.99, *D* = 0.55), FCVpr (*S* > 0.99, *D* = 0.54), GAD (*S* = 0.97, *D* = 0.60), and ISI (*S* = 0.96, *D* = 0.61). Additional directed edges included FCVpr → FCVft (*S* > 0.99, *D* = 0.50), PHQ → GAD (*S* > 0.99, *D* = 0.53), PHQ → ISI (*S* > 0.99, *D* = 0.54), and GAD → ISI (*S* = 0.97, *D* = 0.55). In the men’s network, directed edges included paths from INTRU to AVO (*S* > 0.99, *D* = 0.63), INTRU to FCVpr (*S* > 0.99, *D* = 0.50), and INTRU to GAD (*S* = 0.91, *D* = 0.63). Additional edges were observed from FCVpr → FCVft (*S* > 0.99, *D* = 0.59), GAD → PHQ (*S* > 0.99, *D* = 0.52), and PHQ → ISI (*S* > 0.99, *D* = 0.70). These directed edges reflect conditional dependency relationships meeting the imposed stability thresholds (≥85% selection frequency; ≥50% directional consensus).

**Table 4:** Estimated strength and directional probability among psychological distress variables in the women’s and men’s networks.

Parent node (from)	Descendant node (to)	Women		Men	
		Strength	Direction	Strength	Direction
INTRU^a^	AVO^a^	>0.99	0.59	>0.99	0.63
INTRU^a^	FCVft^b^	>0.99	0.55	—	—
INTRU^a^	FCVpr^b^	>0.99	0.54	>0.99	0.50
INTRU^a^	GAD	0.97	0.60	0.91	0.63
INTRU^a^	ISI	0.96	0.61	—	—
FCVpr^b^	FCVft^b^	>0.99	0.50	>0.99	0.59
PHQ	GAD	>0.99	0.53	—	—
PHQ	ISI	>0.99	0.54	>0.99	0.70
GAD	ISI	0.97	0.55	—	—
GAD	PHQ	—	—	>0.99	0.52

*Note:* FCVft, fear thoughts; FCVpr, physical reactions; PHQ, depressive symptoms; GAD, anxiety symptoms; ISI, insomnia symptoms.

Abbreviations: AVO, avoidance; INTRU, intrusion.

^a^INTRU and AVO are subscales of the IES‐COVID‐19.

^b^FCVft and FCVpr are subscales of the FCV‐19S.

## 4. Discussion

This study modeled the symptom networks of depression, anxiety, insomnia, PTSD, and co‐occurring symptoms in men and women during the 2022 Shanghai COVID‐19 lockdown. To our knowledge, this is the first study to utilize a dual‐network approach, integrating partial correlation and BNs, to map sex differences in psychological distress under acute, large‐scale stress. Unlike most previous studies that only compare sex differences in mental health at the level of intensity or prevalence [21], we specifically addressed critical gaps by exploring associations at the symptom network level in stress‐related symptoms (depression, anxiety, insomnia, fear of COVID‐19, and PTSD) using both partial correlation and BNs. This dual approach allowed us not only to identify which symptoms play a critical role in maintaining network structure and which act as bridges between different constructs but also to uncover potential dynamic causal relationships. The study identified distinct structural configurations among participants grouped by self‐reported sex while recognizing that these patterns may reflect both sex‐related and gender‐related mechanisms.

### 4.1. Sex Differences in Partial Correlation Networks

In our partial correlation network analyses, we observed significant sex differences in the overall network structure. This finding indicates that distress symptoms are organized differently in men and women. Although we initially hypothesized that there would be broad structural similarities, the NCT revealed distinct configurations between men and women under stress [26, 54]. Specifically, these disparities were rooted in centrality patterns. While intrusion and anxiety were the most central nodes in the women’s network, depression played a more dominant role in the men’s network.

A primary contributing factor to these structural differences appears to be the integrative role of pandemic‐related fear within each network. In women, “Fear Thoughts” exhibited significantly greater strength and more extensive associations with other symptoms compared to men. This suggests that for women, the psychological distress during the Shanghai lockdown was more closely anchored to cognitive‐emotional fear, which acted as a central hub interconnecting other symptoms [55]. Conversely, in the men’s network, physical reactions showed relatively stronger connectivity (though not significantly so), hinting at a more somatic‐oriented symptom organization. These findings highlight that women may be more cognitively and emotionally vulnerable to COVID‐19‐specific stressors, which in turn triggers a broader cascade of depression and anxiety symptoms. These divergent patterns likely reflect a complex interplay of sociocultural and biological factors.

In the specific sociocultural context of China—characterized by cultural values that emphasize collective responsibility and emotional restraint [12], the patterns observed among participants grouped by self‐reported sex may partly reflect gender‐related role expectations. As noted in Section 1, women in China often bear a disproportionate burden of domestic and caregiving duties [13]. For the “sandwich generation” of Chinese women, the lockdown intensified work–family conflict and heightened concerns for family safety, leading to greater “cognitive engagement” with the crisis [56]. This sociocultural positioning may explain why fear thoughts emerged as a highly connected hub in women’s networks, strengthening the links between intrusive thoughts and fear‐based cognitions [57, 58]. Indeed, the substantial edge weight between intrusion and fear thoughts in women—contrasted with the weaker association in men—reflects a culturally embedded “cognitive‐to‐cognitive” distress pattern, wherein women’s collective responsibility for family well‐being becomes internalized as persistent cognitive‐emotional processing [59]. For men, the higher centrality of depression, together with its relatively strong descriptive association with insomnia in the men’s network, may resonate with cultural norms of emotional restraint [60]. In Chinese culture, masculine stoicism is socially reinforced, conditioning men to suppress emotional vulnerability [61, 62]. Consequently, psychological pain may manifest as physical symptoms or sleep disturbances rather than overt cognitive distress—a “cognitive‐to‐somatic” or “internalizing” pattern. This somatization pathway is consistent with emerging cross‐cultural research suggesting that sociocultural variables fundamentally influence the topological organization of psychopathological symptoms within network models [14]. Our findings extend this literature by demonstrating how collectivistic orientations, particularly the emphasis on emotional restraint in men, may shape the interconnectivity between mood and somatic symptoms in high‐stress contexts.

Sex‐related biological processes may also be relevant to these divergent patterns. Extensive literature on sex differences in stress reactivity has reported that male stress responses tend to involve the bed nucleus of the stria terminalis (BNST), which is often linked to physiological reactions, whereas female stress responses are more associated with the medial prefrontal cortex (mPFC), a region involved in cognitive processes such as cognitive control [63, 64]. In response to stressors, women tend to experience increased cognitive and emotional distress and show greater cognitive engagement. Consequently, the heightened cognitive engagement in women suggests that cognitive behavioral therapy (CBT), as a process‐based intervention targeting maladaptive thought patterns, could be particularly effective for women in this context [65]. For men, the higher centrality of depression, together with the descriptive prominence of insomnia‐related connections in the men’s network, underscores the need for interventions that address potentially “masked” distress and promote help‐seeking behavior [66, 67].

While these undirected associations provide a structural map of sex‐specific distress, they do not clarify the direction of influence. To address this, we further utilized BN analysis to uncover potential directional dynamics of these symptom interrelations.

### 4.2. Sex Differences in the BNs

While partial correlation networks provide a structural depiction of symptom interrelations, BN analysis further models conditional dependency structures and can therefore offer additional insight into how symptoms are probabilistically related within the overall network. Within this framework, directed edges represent conditional dependencies between nodes, indicating that one symptom may be statistically informative about another, given the rest of the network. These edges should be interpreted as functional dependencies rather than as evidence of temporal or causal relationships. This interpretation is consistent with process‐based therapy (PBT) [68], which emphasizes the functional relations among transdiagnostic processes.

In both sexes, stable directed pathways were identified from Intrusion to avoidance, and from affective symptoms toward insomnia, aligning with previously reported associations [69, 70]. These shared pathways suggest that certain symptom linkages represent common transdiagnostic structures across sexes. Notably, however, the women’s DAG exhibited a more multifaceted configuration, with a greater number of directed edges emerging from intrusion—extending to avoidance, fear thoughts, physical reactions, anxiety, and insomnia. In contrast, the men’s network demonstrated a relatively focused pattern with fewer but more concentrated dependencies, primarily involving depression, anxiety, and insomnia.

Importantly, these directed edges should be interpreted as conditional dependencies within the modeled network rather than definitive causal pathways. From a process‐oriented perspective, however, the differing topological patterns may offer a functional representation of psychological distress that transcends diagnostic categories [71]. Specifically, intrusion appeared more extensively connected within the women’s dependency structure, whereas in men, the affective core, particularly depression and anxiety, was more centrally embedded.

These findings may generate hypotheses for future sex‐sensitive, process‐based intervention research in the context of large‐scale stress. For example, in women, intrusive cognitions may represent a potentially important intervention target warranting further longitudinal and experimental investigation.

However, given the cross‐sectional design inherent to BN analysis, these implications remain exploratory and relate to the issue of ergodicity, meaning that population‐level associations do not necessarily reflect the temporal or causal organization of symptoms within individuals [72]. Therefore, these findings necessitate longitudinal or experimental validation to determine whether intervening on these influential nodes leads to predictable downstream change in individuals.

### 4.3. Strengths and Contributions

The present study offers several significant contributions to the field of psychiatric research. First, by employing a dual‐network approach (GGMs and BNs), it provides a more comprehensive structural map of psychological distress than traditional sum‐score or single‐network methodologies. Second, to our knowledge, this is one of the first studies to move beyond simple prevalence comparisons to explore the transdiagnostic “symptom‐to‐symptom” architecture between sex‐disaggregated groups in a high‐stress, metropolitan lockdown context. Finally, by integrating clinical psychopathology with sociocultural and biological perspectives, the findings provide a nuanced framework for understanding why men and women may require distinct therapeutic pathways even when exposed to the same large‐scale stress conditions.

### 4.4. Limitations

Despite its strengths, several limitations warrant caution. First, reliance on self‐reported data from online questionnaires may introduce a recall bias and social desirability effects. Second, the cross‐sectional design precludes definitive conclusions about temporal causality. While BNs provide plausible directional hypotheses, they must be validated by longitudinal designs. Third, the DAG analyses in this study were primarily exploratory, focusing on identifying potential sex‐specific pathways among stress‐related symptoms rather than conducting formal statistical comparisons of network structures. Consistent with the established role of DAGs in the discovery of pathways and generating hypotheses about directional dependencies, we descriptively examined stable, bootstrap‐robust arcs (5000 iterations) to highlight comparative patterns and candidate symptom pathways without inferential testing. Notably, formal statistical comparisons of DAGs across groups (e.g., via differential causal inference methods) [73] typically require assumptions such as a shared topological order between the networks, which may not hold when group‐specific effects substantially alter causal ordering. Given these limitations and the exploratory aims of DAG analysis, a descriptive approach was deemed more appropriate. In addition, the symptom domains appear to be interrelated but with distinct probabilistic dependencies in the DAGs. The minimal influence of certain symptoms on others is noteworthy given their recognized relevance in stress‐related psychopathology. However, it is important to emphasize that BNs model probability distributions rather than establishing true causal links [31, 74]. Thus, the directed edges identified indicate probabilistic dependencies (e.g., intrusion predicting fear thoughts in women) without implying causality. Future research might aim to identify causal relationships by combining longitudinal and experimental approaches, ideally paired with interventions.

Fourth, the measurement instruments used in this study are an additional limitation. Some of the questionnaires, including the IES, were developed based on DSM‐IV diagnostic criteria and do not fully align with DSM‐5 symptom classifications. Specifically, the IES does not comprehensively assess the negative alterations in cognition and mood cluster introduced in the DSM‐5 PTSD criteria. Consequently, certain symptom domains may not have been captured in the present network models, which could influence the observed network structure and centrality patterns. Fifth, although participants were grouped according to self‐reported sex, the study did not separately assess gender identity, gender roles, caregiving burden, occupational role expectations, or other gender‐related social exposures. Therefore, the observed differences between women and men should be interpreted as sex‐disaggregated findings that may reflect both sex‐related biological processes and gender‐related sociocultural mechanisms. Finally, the sample was drawn from adults in Shanghai during the uniquely stringent 2022 lockdown, which may limit the study’s generalizability to other crisis contexts or cultural settings.

## 5. Conclusion

This study highlights significant differences between women and men in the structure and potential directional interplay of stress‐related distress symptoms among adults under acute pandemic stress. Our findings underscore the necessity of moving beyond traditional categorical comparisons to consider sex‐sensitive network topographies when conceptualizing psychopathology.

The results suggest that for women, distress symptoms were more densely interconnected, with anxiety and intrusion occupying relatively central positions and fear‐related cognitions showing broad connectivity. Conversely, for men, the system appears more centralized around depression and its strong associations with somatic markers like insomnia. These divergent configurations imply that a “one‐size‐fits‐all” approach to mental health may be insufficient during large‐scale crises. Effective crisis management must involve a paradigm shift toward personalized, sex‐ and gender‐sensitive frameworks that address the specific process of distress unique to each group.

## 6. Practical Implications

The present findings offer conceptual and actionable insights that may inform public health policy by advocating for a paradigm shift toward sex‐ and gender‐sensitive mental health strategies. The sex‐disaggregated network structures identified here suggest that women and men may experience and organize distress through partially distinct pathways. These structural differences, while group‐level observations, provide a conceptual framework for tailoring prevention and early intervention efforts. In this light, understanding these sex‐related configurations can strengthen the conceptual foundation for prevention and early detection frameworks, supporting a shift away from traditional, category‐based diagnostic protocols toward process‐based, sex‐sensitive mental health strategies that target specific functional interrelations among symptoms at the population level [75]. Public health strategies may benefit from being informed by influential group‐specific “hub symptoms”: for women, population‐level patterns suggest that distress may be more closely organized around cognitive‐emotional regulation processes [68, 71], which might be addressed with CBT techniques addressing intrusive thoughts and fear reappraisal; for men, practitioners should remain alert to “masked” or somaticized distress, emphasizing depressive symptoms and physiological regulation through sleep hygiene or biofeedback targeting somatic arousal. From a public health perspective, screening tools should transition from aggregate scores to the identification of influential nodes, such as intrusive memories in women and sleep disturbances in men, to facilitate the early detection of individuals at risk for system‐wide symptom cascades. Furthermore, policy‐makers should leverage urban technological infrastructures, like those in Shanghai, to provide low‐threshold, digitally delivered, and cross‐diagnostic support during future crises [10]. By fostering sex‐sensitive and integrated mental health services, these tailored approaches can more effectively mitigate psychological distress in diverse populations facing public health emergencies.

## Author Contributions

**Jingchu Hu**: conceptualization, methodology, investigation, funding acquisition, supervision, writing – original draft, writing – review and editing, reanalysis of the data, regeneration of figures, and preparation of reproducible analysis code during revision. **Yiting Huang**: data curation, formal analysis, visualization, project administration. **Marlon Westhoff**: methodology, validation, writing – review and editing. **Qingzhuo Ren**: initial investigation. **Stefan G. Hofmann**: conceptualization, supervision, writing – review and editing.

## Funding

This study was supported by the National Natural Science Foundation of China (Grant 82201672), the Youth Foundation Project of Humanities and Social Sciences from Chinese Ministry of Education (Grant 22YJC190008) to Jingchu Hu, the Shenzhen Fundamental Research Program (Grant JCYJ20230807142359032) to Jingchu Hu, and the Shenzhen Fund for Guangdong Provincial High‐level Clinical Key Specialties (Grant SZGSP013).

## Disclosure

The funders had no role in the study design, data collection, data analysis, interpretation of the findings, preparation of the manuscript, or decision to submit the manuscript for publication. Dr. Hofmann receives financial support by the Alexander von Humboldt Foundation (as part of the Alexander von Humboldt Professur), the Hessische Ministerium für Wissenschaft und Kunst (as part of the LOEWE Spitzenprofessur), the DYNAMIC center, funded by the LOEWE program of the Hessian Ministry of Science and Arts (Grant LOEWE1/16/519/03/09.001(0009)/98), the Deutsche Forschungsgemeinschaft (DFG, German Research Foundation) – Project‐ID 521379614 – TRR 393SFB/Transregio 393 and the Excellence Cluster EXC3066 “The Adaptive Mind” (Project No. 533717223). He also receives compensation for his work as editor from the American Psychological Association and royalties and payments for his work from various publishers.

## Conflicts of Interest

The authors declare no conflicts of interest.

## Supporting Information

Additional supporting information can be found online in the Supporting Information section.

## Supporting information


**Supporting Information** Table S1: Regularized partial‐correlation edge‐weight matrix for the women’s symptom network. Table S2: Regularized partial‐correlation edge‐weight matrix for the men’s symptom network. Figure S1: Statistical power analysis. Figure S2: Accuracy of edge‐weight estimates in the women’s network assessed using nonparametric bootstrapping. Figure S3: Accuracy of edge‐weight estimates in the men’s network assessed using nonparametric bootstrapping. Figure S4: Bootstrapped edge‐weight difference test for the women’s network. Figure S5: Bootstrapped edge‐weight difference test for the men’s network. Figure S6: Stability of strength centrality estimated using case‐dropping bootstrapping for the women’s network. Figure S7: Stability of strength centrality estimated using case‐dropping bootstrapping for the men’s network.

## Data Availability

The data and analysis code that support the findings of this study are openly available in the Open Science Framework (OSF) at https://osf.io/tdv8u/.
