# Supplementary material for: Mapping Sex Differences in Depression, Anxiety, and Co‐Occurring Symptoms in Adults Under Stress: A Bayesian Network Approach
Source: Depress Anxiety. 2026 Sep 12;2026:6640438. doi: 10.1155/da/6640438 (PMC13570569; doi:10.1155/da/6640438)
Supplement: Supplementary file 1 [file DA-2026-6640438-s001.docx]

**Mapping Sex Differences in Depression, Anxiety, and Co-occurring Symptoms in Adults Under Stress: A Bayesian Network Approach**

*Supplementary Materials*

**Supplementary Table 1.** Regularized partial-correlation edge-weight matrix for the women's symptom network.

| Variables | **INTRU** | **AVO** | **FCVft** | **FCVpr** | **PHQ** | **GAD** | **ISI** |
| --- | --- | --- | --- | --- | --- | --- | --- |
| INTRU ^a^ | — |  |  |  |  |  |  |
| AVO ^a^ | 0.38 | — |  |  |  |  |  |
| FCVft ^b^ | 0.20 | 0.11 | — |  |  |  |  |
| FCVpr ^b^ | 0.17 | 0.10 | 0.52 | — |  |  |  |
| PHQ | 0 | 0 | 0 | 0 | — |  |  |
| GAD | 0.14 | 0.06 | 0.05 | 0 | 0.61 | — |  |
| ISI | 0.15 | 0 | 0 | 0 | 0.30 | 0.18 | — |

Note. INTRU = Intrusion subscale of the Impact of Event Scale modified for COVID-19 (IES-COVID-19); AVO = Avoidance subscale of the IES-COVID-19; FCVft = Fear Thoughts subscale of the Fear of COVID-19 Scale (FCV-19S); FCVpr = Physical Reactions subscale of the FCV-19S; PHQ = depressive symptoms assessed using the Patient Health Questionnaire-9; GAD = anxiety symptoms assessed using the Generalized Anxiety Disorder-7; ISI = insomnia symptoms assessed using the Insomnia Severity Index. Values represent regularized partial-correlation edge weights. Diagonal elements are omitted.

**Supplementary Table 2.** Regularized partial-correlation edge-weight matrix for the men's symptom network.

| **Variables** | **INTRU** | **AVO** | **FCVft** | **FCVpr** | **PHQ** | **GAD** | **ISI** |
| --- | --- | --- | --- | --- | --- | --- | --- |
| INTRU ^a^ | — |  |  |  |  |  |  |
| AVO ^a^ | 0.42 | — |  |  |  |  |  |
| FCVft ^b^ | 0 | 0.08 | — |  |  |  |  |
| FCVpr ^b^ | 0.32 | 0 | 0.51 | — |  |  |  |
| PHQ | 0 | 0 | 0 | 0 | — |  |  |
| GAD | 0.13 | 0 | 0.08 | 0 | 0.63 | — |  |
| ISI | 0 | 0 | 0 | 0 | 0.42 | 0.09 | — |

Note. INTRU = Intrusion subscale of the Impact of Event Scale modified for COVID-19 (IES-COVID-19); AVO = Avoidance subscale of the IES-COVID-19; FCVft = Fear Thoughts subscale of the Fear of COVID-19 Scale (FCV-19S); FCVpr = Physical Reactions subscale of the FCV-19S; PHQ = depressive symptoms assessed using the Patient Health Questionnaire-9; GAD = anxiety symptoms assessed using the Generalized Anxiety Disorder-7; ISI = insomnia symptoms assessed using the Insomnia Severity Index. Values represent regularized partial-correlation edge weights. Diagonal elements are omitted.

**Supplementary Figure 1.** Statistical power analysis.

Power analysis was conducted using the R package powerly with 50 simulation replications at each of 30 candidate sample sizes for a Gaussian graphical model with seven nodes, assumed density of 0.40, target sensitivity of 0.60, and target power of 0.80. Spline uncertainty used the package default of 10,000 bootstrap runs. The recommended sample size was 168 (95% interval: 150–187), lower than the sample size in both women (n = 684) and men (n = 507).

**Supplementary Figure 2.** Accuracy of edge-weight estimates in the women's network assessed using nonparametric bootstrapping. Red points connected by a red line represent original-sample edge weights, black points represent bootstrap means, and gray intervals represent bootstrapped 95% confidence intervals. Narrower intervals indicate greater precision.

**Supplementary Figure 3.** Accuracy of edge-weight estimates in the men's network assessed using nonparametric bootstrapping. Red points connected by a red line represent original-sample edge weights, black points represent bootstrap means, and gray intervals represent bootstrapped 95% confidence intervals. Narrower intervals indicate greater precision.

**Supplementary Figure 4.** Bootstrapped edge-weight difference test for the women's network. Black cells indicate pairs of edge weights that differed significantly at alpha = 0.05; gray cells indicate nonsignificant differences. Colored diagonal cells represent estimated edge weights, with blue indicating positive and red negative associations.

**Supplementary Figure 5.** Bootstrapped edge-weight difference test for the men's network. Black cells indicate pairs of edge weights that differed significantly at alpha = 0.05; gray cells indicate nonsignificant differences. Colored diagonal cells represent estimated edge weights, with blue indicating positive and red negative associations.

**Supplementary Figure 6.** Stability of strength centrality estimated using case-dropping bootstrapping for the women's network.

**Supplementary Figure 7.** Stability of strength centrality estimated using case-dropping bootstrapping for the men's network.
